# An Open Letter

**Published:** 1881-04

**Authors:** T. F. Pomeroy

**Affiliations:** Jersey City


### AN OPEN LETTER.

Ed. Homœopathic Physician.—In your February number I notice under the title—that old familiar one—"Fatal Errors," the following question propounded by its author, my esteemed friend, Dr. Ad. Lippe, viz.: "Is it not also a fatal error if the adherents of Hahnemann's strict inductive method, endorse eclectic journals, such as publish eclectic papers, by writing truly homœopathic papers for them?" I answer unhesitatingly, No! As well might it be asked, shall education and *instruction* cease? Those who are well need not the physician, only the sick; and only the ignorant, as to any particular subject, need instruction in it, surely not those who are already—as to homœopathy—adherents of, and therefore presumably experts in, "Hahnemann's strict inductive method."

"It would have been a *fatal error* had the members of the International Hahnemann Association separated themselves from the American Institute of Homœopathy." So, most emphatically, it would and wherefore? In reply to this, another question propounded by the doctor in the paper referred to, I can not accept, though *he* must, to be consistent, the reason that he urges against writing for such journals "as publish eclectic papers." When it can be made to appear that there are not many eclectics within the membership of the American Institute of Homœopathy, and that its "Transactions" are in no degree tinctured with eclecticism or its proceedings, government and control in no manner influenced thereby, *then* only, can it be *consistently* affirmed that it would have been a fatal error to have thus separated from the Institute. By retaining their membership in the American Institute of Homœopathy, the founders of the International Hahnemann Association acted most wisely in recognizing thereby an ability to influence that body to a higher appreciation of "Hahnemann's strict inductive method"; as also to a stricter conformance thereto in their practice. This action is in harmony with the universal idea, and the uniform purposes of education. This is the unquestionable object of all educational processes; and that the American Institute of Homœopathy stands greatly in need of instruction in the inductive method of Hahneman, requires no farther demonstration than an attencence upon one of its sessions or a perusal of many, very many, of the papers furnished by its members to our numerous medical journals, if not also in many appearing in its "Transactions." Surely, then, if the great American Institute of Homœopathy needs instruction and *endorsement* at the hands of experts in the inductive method of Hahnemann, how much more so do these otherwise forsaken and cast-off eclectics and their journals need such enlightenment? Moreover, if we may not write "homœopathic papers for *them*," who shall tell us for what journals we may write such papers without the fear of writing for those that are not more or less eclectic, or heterodox, as to "Hahnemann's strict inductive methods"; who will undertake to point out which of our journals are orthodox as to those methods, and which are not?

Where, I ask, would homœopathy and its *truly strict* inductive methods have been to-day had not the eclectics and the allopaths and whosoever would, been invited to come and drink of its pure waters? How can ignorance, general or particular, be removed but through enlightenment and instruction? and should not that enlightenment be most freely accorded when the darkness is greatest, and instruction most freely given when it is most needed? Most assuredly so. Hahnemann's strictly inductcive methods are no exception to this rule; let whosoever will ome and partake of these living waters freely, most fully; even, the despised and greatly contemned eclectics; and let them be invited to come, and urged to come, and convinced, so that they will come, if it does involve the necessity of writing papers for publication in their journals. "No pent up Utica contracts our powers, the whole boundless continent is ours"; but ours only through a persistent, a determined effort to *judiciously* inculcate and disseminate the precepts and the practices taught through "Hahnemann's strictly inductive methods."

T. F. POMEROY.

Jersey City, Feb. 15th, 1881.

---

